# ATF5, a putative therapeutic target for the mitochondrial DNA 3243A > G mutation-related disease

**DOI:** 10.1038/s41419-021-03993-1

**Published:** 2021-07-14

**Authors:** Xinpei Gao, Zhixin Jiang, Xinfeng Yan, Jiping Liu, Fengwen Li, Peng Liu, Jialu Li, Yuehua Wei, Yi Eve Sun, Yinan Zhang, Congrong Wang

**Affiliations:** 1 Shanghai Institute of Stem Cell Research and Clinical Translation, Shanghai East Hospital, Tongji University, School of Medicine, Shanghai, China; 2 Shanghai Jiao Tong University Affiliated Sixth People’s Hospital, Shanghai Key Laboratory of Diabetes, Department of Endocrinology and Metabolism, Shanghai, China; 3 Shanghai East Hospital, Tongji University School of Medicine, Department of Endocrinology, Shanghai, China; 4 Department of Oncology, Shanghai Ninth People’s Hospital, Shanghai Jiao Tong University School of Medicine, Shanghai, People’s Republic of China; 5 Shanghai Jiao Tong University Affiliated Sixth People’s Hospital, The Metabolic Disease Biobank, Shanghai, China; 6 Department of Endocrinology & Metabolism, Shanghai Fourth People’s Hospital, School of Medicine, Tongji University, Shanghai, China

**Keywords:** Mechanisms of disease, Diabetes

## Abstract

The mitochondrial DNA m.3243A > G mutation is well-known to cause a variety of clinical phenotypes, including diabetes, deafness, and osteoporosis. Here, we report isolation and expansion of urine-derived stem cells (USCs) from patients carrying the m.3243A > G mutation, which demonstrate bimodal heteroplasmy. USCs with high levels of m.3243A > G mutation displayed abnormal mitochondrial morphology and function, as well as elevated ATF5-dependent mitochondrial unfolded protein response (UPR^mt^), together with reduced Wnt/β-catenin signaling and osteogenic potentials. Knockdown of ATF5 in mutant USCs suppressed UPR^mt^, improved mitochondrial function, restored expression of *GSK3B* and *WNT7B*, and rescued osteogenic potentials. These results suggest that ATF5-dependent UPR^mt^ could be a core disease mechanism underlying mitochondrial dysfunction and osteoporosis related to the m.3243A > G mutation, and therefore could be a novel putative therapeutic target for this genetic disorder.

## Introduction

Mitochondrion is the center for energy production and its dysregulation has been linked to various human diseases [1–3]. By metabolizing glucose and lipids through TCA cycle and β-oxidation, respectively, mitochondria store biological energy in adenosine triphosphate (ATP). This process requires the coordination of more than 80 proteins that form 5 major respiratory chain complexes, thirteen of which are encoded by the mitochondrial genome [4, 5]. In addition to the 13 respiratory chain subunits, the mitochondrial genome also encodes 2 rRNAs and 22 tRNAs, mutations of which have been reported to be involved in certain diseases, such as Leigh syndrome, Kearns-Sayre syndrome, Lever hereditary optic neuropathy (LHON), etc. [6, 7]. The mitochondrial DNA A3243G (m.3243A > G) mutation in the *tRNA*^*Leu (UUR)*^ gene is one of the most common mutations in the mitochondrial genome, which is linked to a clinical syndrome termed MELAS (mitochondrial encephalo-myopathy, lactic acidosis, and stroke-like episodes) [8, 9]. The m.3243A > G mutation is also associated with other clinical features, including maternally inherited sensorineural hearing impairment, as well as diabetes, accompanied by other phenotypes, such as, cardiomyopathy, ataxia, basal ganglia calcification, and macular retinal dystrophy [10]. Interestingly, a previous case-control study indicated that the m.3243A > G mutation is associated with premature bone aging, characterized by reduced bone mass, impaired structure and strength [11]. Our recent study further suggested that high levels of the m.3243A > G mutation in blood leukocytes were significantly associated with lower bone mineral density [12].

Such a broad spectrum of clinical manifestations could be attributed to the heteroplasmic nature of the m.3243A > G mutation, where mutant transcripts could present in different ratio to wild-type transcripts in different cell types or tissues [13–15]. Biochemical analysis revealed that high heteroplasmy levels of the m.3243A > G mutation reduced mitochondrial tRNA^Leu (UUR)^ abundance, decreased its aminoacylation, and thus inhibited normal post-transcriptional modifications [16–20]. Cells with high levels of the m.3243A > G mutation have impaired protein synthesis and respiratory activity [21, 22]. However, the molecular mechanism underlying the pathology of osteogenesis remains unclear.

Recently, the mitochondrial unfolded protein response (UPR^mt^) has been found to protect mitochondria from damages caused by misfolded or mutated proteins [4, 23–26]. Loss of mitochondrial DNA or imbalance between mitochondria-nuclear protein synthesis activates UPR^mt^, which in turn improves mitochondrial function and promotes survival in *C. elegans* [27, 28]. ATF5 is a transcription factor functions in the UPR^mt^ pathway. It targets genes involved in mitochondrial protein homeostasis [29, 30], including *mtHSP70*, *HSP60* and *LONP1*. In contrast to this positive role, in *C. elegans*, constitutively active UPR^mt^ has been reported to increase mutated mitochondrial DNA, leading to mitochondrial dysfunction [31, 32].

To examine the molecular etiology underlying the mitochondrial disorder caused by the m.3243A > G mutation, we generated urine-derived stem cells (USCs) from patients with the m.3243A > G mutation. We reported that high levels of m.3243A > G heteroplasmy activated ATF5-dependent UPR^mt^, which in turn inhibited mitochondrial functions, the m.3243A > G mutation simultaneously inhibited the Wnt/β-catenin pathway, which is known to be essential for osteogenesis [33]. Downregulation of UPR^mt^ by ATF5 knockdown rescued the defects in mitochondrial function, disinhibited Wnt/β-catenin, and consequently improved osteogenesis from mutant USCs. Our study provided the first connection between UPR^mt^ and osteogenesis, suggesting a possible mechanism underlying the pathology of m.3243A > G related osteoporosis, and identified ATF5 as a potential therapeutic target for this disease.

## Results

### Clinical characteristics of patients with the m.3243A > G mutation

**Fig. 1:**
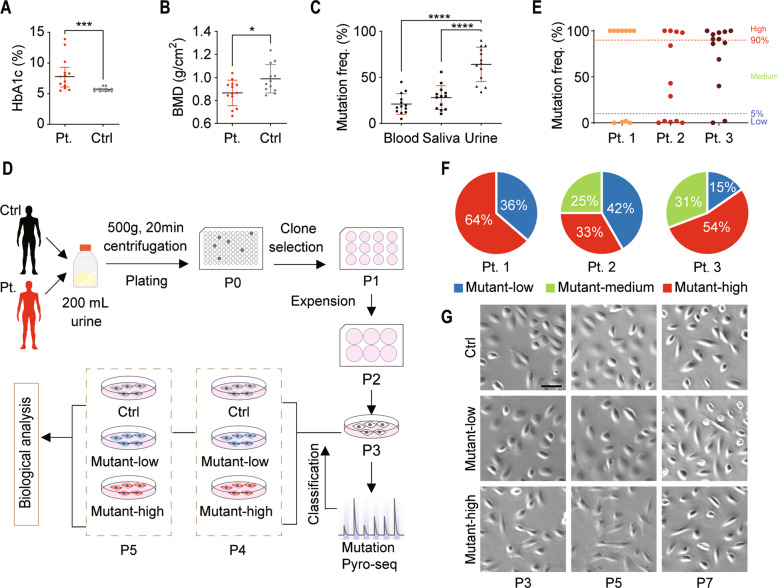
Isolation and proliferation of urine-derived mesenchymal stem cells (USCs) from patients with mitochondrial DNA 3243A > G mutation and health controls. Box plots of HbA1c levels (**A**) and BMD T-score at total hip (**B**) in 13 m.3243A > G mutation carriers and 13 age, sex, BMI-matched healthy controls. **C** Box plot of m.3243A > G mutation frequency in leukocytes, saliva and urine sediment of 13 m.3243A > G mutation carriers. **D** A schematic workflow of isolation urine-derived stem cells from urine. Urine samples were collected from patients with mt.3243A > G mutation and healthy controls and then centrifuged. Cells in the urine sample were resuspended in fresh USC medium, plated on 96-well plated coated with gelatin for 7 days. Colonies were identified and sub-cultured in 12-well plate, 6-well plate and 10 cm plate to expand. The levels of heteroplasmy in the derived USC clones were evaluated by pyrosequencing at passage 3 and marked for further analysis. **E** The USCs showed a bimodal degree of mutation heteroplasmy, e.g., either high (>90%) or low (<5%) m.3243A > G contents, with only few clones containing medium level of mutation from three m.3243A > G mutation carriers. **F** The distribution of mutant-low, mutant-medium and mutant-high USC clones from three m.3243A > G mutation carriers. **G** USCs retained a robust proliferation capability after several passages. No differences in cell morphology were found in USCs with different heteroplasmy levels. Scale bar = 50 µm.

A total of 13 patients (8 males and 5 females) with the m.3243A > G mutation as well as 13 age, sex, BMI-matched healthy controls were enrolled in this study. Clinical characteristics of all subjects were shown in supplementary Table S1. Compared to controls, the median levels for HbA1c were significantly higher in the m.3243A > G mutation group (7.8% vs. 5.7%, *P* < 0.01) (Fig. 1A and Table S1). Twelve out of 13 m.3243A > G carriers were diagnosed with diabetes mellitus (92.3%). The mean age at diagnosis was 40.75 ± 14.50 years, and mean diabetes duration was 6.67 ± 7.62 years, which were typical for patients with this genetic disorder. As expected, another typical feature of this disease, bilateral sensorineural hearing loss was observed in 8 out of 13 patients (61.54%), In addition to these two well-known clinical manifestations, a high proportion (53.85%) of m.3243A > G patients were diagnosed with osteoporosis/osteopenia. Bone mineral densities (BMD) of total hip and femoral stem in mutation carriers were significantly lower than those of controls (total hip: 0.87 ± 0.11 vs. 0.99 ± 0.12 g/cm^2^, femoral stem: 1.04 ± 0.13 vs. 1.18 ± 0.16 g/cm^2^; *P*_s_ < 0.05) (Fig. 1B and Table S1), which was consistent to previous reports [11] demonstrating osteoporosis being a novel disease related phenotype. Among many other possibilities, the decreased BMD was most likely resulted from deficits in osteogenic differentiation from mesenchymal stem cells (MSCs).

### Isolation and characterization of USCs from controls and patients with the m.3243A > G mutation

The average heteroplasmy levels of the m.3243A > G mutation in leukocytes, saliva, and urine sediment were 21.15 ± 11.15%, 28.06 ± 12.93% and 64.24 ± 18.81%, respectively (Table S1 and Fig. 1C), indicating that urine sediment was enriched for the m.3243A > G mutation. USCs are mainly composed of MSC-like cells, which can self-renew and differentiate to osteocytes, chondrocytes, as well as adipocytes [34]. Patients-specific USCs could be an ideal source for studying the cellular and molecular mechanisms underlying m.3243A > G mutation-related diseases, particularly, osteoporosis.

We derived USCs from three healthy individuals and three patients with the m.3243A > G mutation, whose detailed clinical information was presented in Table 1. USCs were isolated from urine samples by minimal processing as shown in Fig. 1D. After 5–7 days of the initial plating, small, compact cell clusters derived from individual cells were observed (Passage 0, P0). These cells began to form larger colonies after 7 days of additional culturing (P1, Fig. 1D). We were able to isolate and expand 11, 12 and 13 USC clones from Patient 1, 2, and 3, respectively. The levels of heteroplasmy in these patient-specific USC clones were evaluated by pyrosequencing at P3 (Fig. 1D, E and S1). The USC clones showed a bimodal distribution of mutation heteroplasmy, e.g. either high (>90%) or low (<5%) in m.3243A > G contents, with only a few clones containing medium levels of the mutation (Fig. 1E, F). We termed USC clones with high mutation rates (>90%) as “Mutant-high (Mut-H)”, and low mutation rates (<5%) as “Mutant-low (Mut-L)”. The morphologies of USC clones, whether or not carrying the genetic mutation, were uniformly spindle-like, characteristic of MSCs. These USCs retained robust proliferation capabilities after several passages (Fig. 1G). The heteroplasmy levels of USCs remained the same for at least 7 passages, suggesting a stable transmission of the mutation (Table S2 and S3). The m.3243A > G mutation did not appear to alter the karyotype of the USCs (SI Fig. S2). Flow cytometry revealed that all USC clones from affected patients and controls showed stable expression of SSEA-4 (SI Fig. S3), as well as other MSC cell surface markers (CD29, CD73, CD90), renal epithelial marker (CD13), and epithelial basal cell marker (CD44). As expected, the USCs were negative for hematopoietic stem cell markers (CD34, CD45), endothelial lineage markers (CD31), and human leukocyte antigen (HLA-DR). These data indicated that these cells were originated from renal tissues, rather than from hematopoietic or endothelial lineages.

**Table 1:** Information on patient donors with m.3243A > G and healthy controls for generation of USCs.

	Age (years)	Sex	BMI (kg/m^2^)	Typical clinical features	BMD of total hip (g/cm^2^)	HbA1c (%)	Medication
Patient 1	31	Male	18.5	Diabetes mellitus; SNHL;	0.918	8.0	Insulin 34 U/day
Patient 2	30	Male	17.9	Diabetes mellitus; SNHL	0.843	5.9	Insulin 30 U/day; acarbose 50 mg/day
Patient 3	24	Male	20.1	Diabetes mellitus; SNHL	0.726	13	Insulin 60 U/d; acarbose 300 mg/day
Healthy control 1	30	Male	21.6	/	0.978	5.4	/
Healthy control 2	39	Male	20.4	/	0.888	5.8	/
Healthy control 3	27	Male	19.0	/	0.861	5.2	/

### Impaired mitochondrial morphology and function in mutant-high USCs

**Fig. 2:**
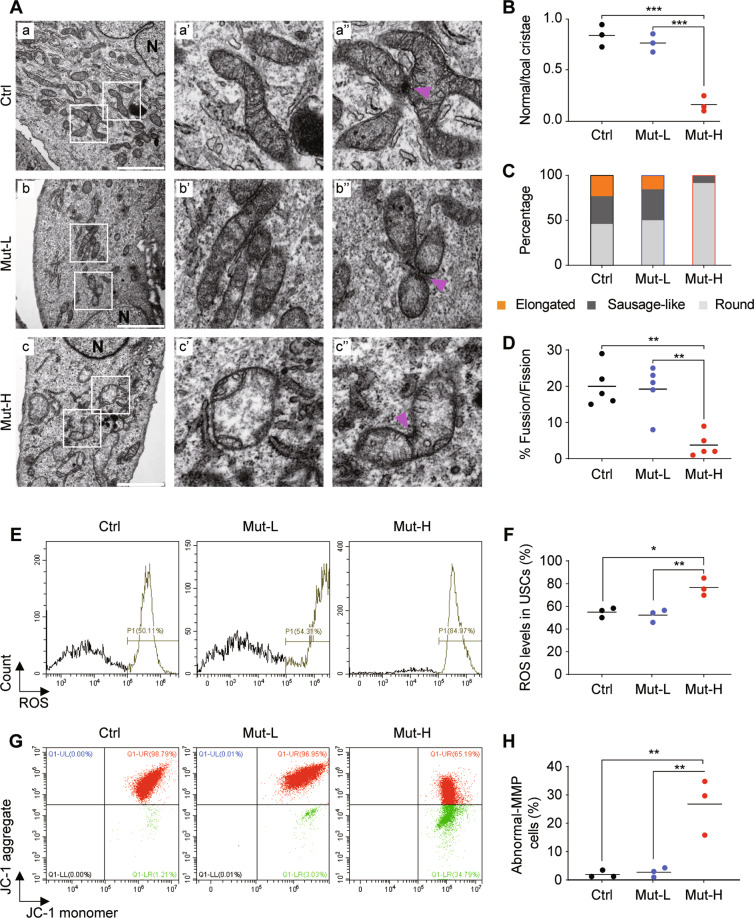
Impaired mitochondrial morphology and function in m.3243A > G mutant-high USCs. **A** Mitochondrial morphology as examined by transmission electric microscopy (TEM). Mitochondrial morphology in Ctr USCs (a–a”: a. Cross-section of Ctr USCs showed round, sausage-like and elongated mitochondria; a’. Enlarged mitochondrial with rich cristae; a”. Mitochondrial junction indicating fission/fusion events (purple arrow)), Mut-L USCs (b–b”, similar to those in controls), and Mut-H USCs (c–c”: c. cross-section of Mut-H USCs showed mostly abnormally round, little sausage-like and no elongated mitochondria; c’. Enlarged mitochondria lacking normal cristae; and c”. abnormal mitochondrial junctions). **B** Quantification plot showed Mut-H USCs contained high levels of mitochondria with impaired cristae structure. **C** Quantification plot showed Mut-H USCs contained less elongated and sausage-like mitochondria. **D** Quantification plot showed Mut-H USCs contained less fission/fusion junctions. **E** Intracellular reactive oxygen species (ROS) generation was measured by flow cytometry detecting DCF fluorescence intensity. **F** Quantification plot for **E**. The ROS level was increased in Mut-H USCs. **G** The percentage of abnormal-MMP cells was measured by flow cytometry detecting the percentage of green fluorescence of JC-1 dye. **H** Quantification plot for **G**. The mitochondrial membrane potential was decreased in Mut-H USCs USCs. Scale bar: 6 µm. Mitochondria with indicated morphologies were quantified from TEM images. *P < 0.05, **P < 0.01, ***P < 0.001.

The bimodal segregation and stable transmission of the mtDNA mutation heteroplasmy provided us an ideal isogenic setting for further analyses. We examined the mitochondrial morphology of patient-specific USCs with high and low mutations as well as controls by using transmission electron microscopy (TEM). As shown in Fig. 2, mitochondria of control and Mut-L USCs were enriched with normal cristae (Fig. 2A–a, b; a’, b’; a”, b”). However, mitochondria of Mut-H USCs displayed abnormal cristae structures (Fig. 2A–c, c’, c”, 2B). Healthy mitochondria displayed dynamically connected tubular structures, which were elongated and sausage-like (Fig. 2A–a), while Mut-H USCs contained much less elongated and sausage-like mitochondria (Fig. 2C). Instead, mitochondria of Mut-H USCs appeared swollen and with decreased matrix density. From TEM images, we also observed connected mitochondria, indicative of fission or fusion events (Fig. 2A, arrow heads). The percentage of mitochondria undergoing fission/fusion in Mut-H USCs was significantly lower than those in Mut-L and control USCs (Fig. 2D). Together, these results demonstrated that mitochondrial morphology was significantly impaired by high but not low levels of m.3243A > G mutation.

Mitochondrial functions were further examined by reactive oxygen species (ROS) and mitochondrial membrane potentials (*ΔΨ*_*m*_). By staining cells with DCFH-DA, a well-established marker for intracellular ROS, we showed that Mut-H USCs had higher levels of ROS, as compared to Mut-L and control USCs (Fig. 2E, F). In addition, by staining USCs with JC-1, we found that Mut-H USCs had much lower *ΔΨ*_*m*_ than Mut-L and control USCs (Fig. 2G, H).

### High M.3243A > G heteroplasmy levels activated UPR^mt^ and reduced Wnt/β-catenin signaling in USCs

**Fig. 3:**
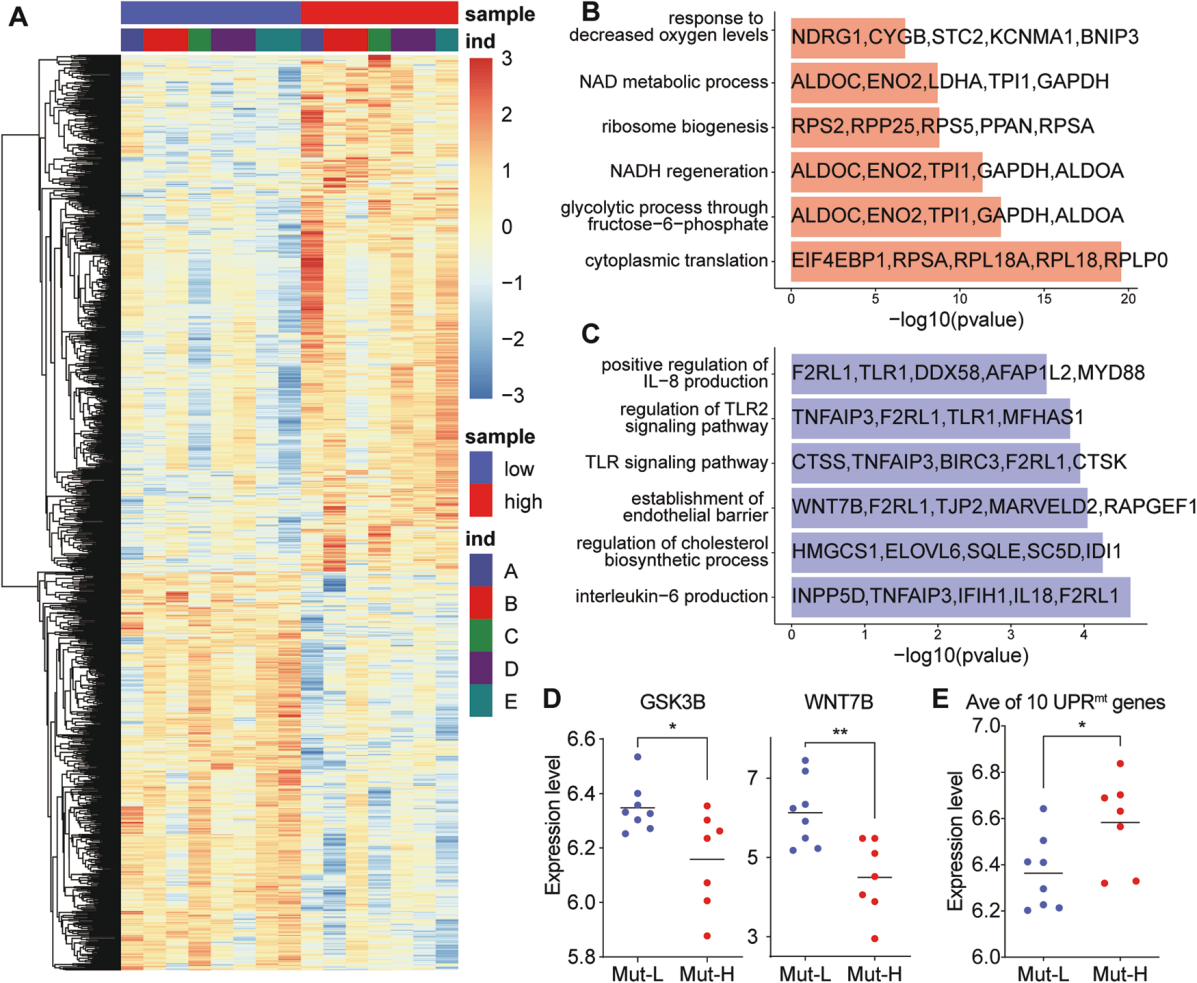
Different transcriptional profiles between Mut-H and Mut-L USCs. **A** Heatmap of whole transcriptome data showing different gene transcription from Mut-H to Mut-L USCs. **B** Gene ontology (GO) analysis exhibit different enrichment of gene families in Mut-H USCs. **C** Gene ontology (GO) analysis exhibit different enrichment of gene families in Mut-L USCs. **D** GSK3B and WNT7B mRNA expression decreased in Mut-H USCs. **E** A panel of ten genes related to UPR^mt^ was found to express higher in Mut-H USCs. *P < 0.05, **P < 0.01.

**Fig. 4:**
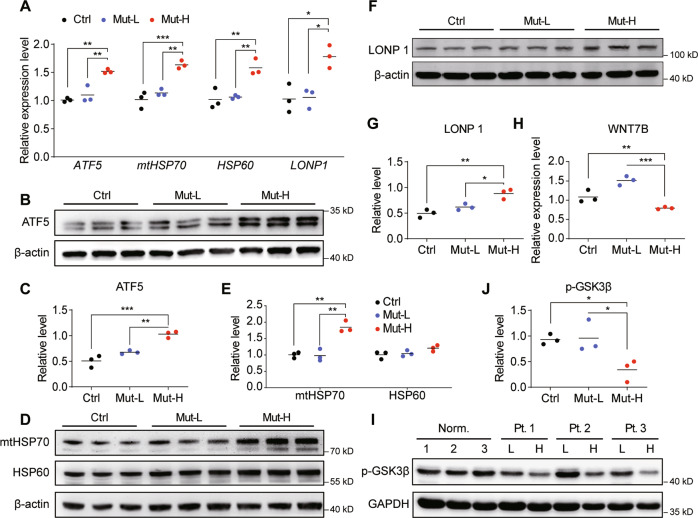
Mut-H USCs had elevated UPR^mt^ and reduced GSK3B and WNT7B. **A** The mRNA levels of UPR^mt^ target genes (*ATF5, HSP70, HSP60 and LONP1*) were elevated in Mut-H USCs. Total RNAs were extracted from Ctr, Mut-L and Mut-H USCs, and expression of UPR^mt^ target genes were quantified through real-time quantitative PCR (RT-qPCR). **B**–**G** The protein levels of UPR^mt^ target genes (*ATF5, HSP70, HSP60 and LONP1*) were elevated in mutant-high USCs. Total protein lysates were extracted from USCs and protein levels were analyzed by western blot. β-actin served as internal loading control. **C**, **E**, **G** were quantification plot for **B**, **D**, **F**, respectively. **H** mRNA levels of WNT7B were reduced in Mut-H USCs. **I** Protein levels of p-GSK3β were decreased in Mut-H USCs. GADPH serves as internal control. **J** Quantification plot for **I**. *P < 0.05, **P < 0.01, ***P < 0.001.

RNA sequencing was performed to determine the molecular basis of the morphological and physiological changes between Mut-H and Mut-L USCs (Fig. 3A). Gene ontology (GO) analysis exhibited enriched expression of several gene families in Mut-H versus Mut-L USCs (Fig. 3B, C). Genes in NAD^+^/NADH metabolic pathway as well as genes in response to oxygen levels, which typically regulate mitochondrial functions, were affected (Fig. 3B, C). Interestingly, a potent Wnt ligand, *WNT7B* as well as a core Wnt signaling component, *GSK3B*, were down-regulated in Mut-H USCs (Fig. 3D), consistent with the notion that Mut-H USCs had reduced osteogenic potentials. Since impaired mitochondrial function could induce UPR^mt^, we also examined a panel of 10 UPR^mt^ genes (*ATF5, HSPA4, HSPD1, LONP1, DDIT3, SPG7, HSPA9, DNAJA3, CLPP, ATF4*), and found out that, the average expression level of these genes was elevated in Mut-H USCs (Fig. 3E). We confirmed this result by RT-qPCR (Fig. 4A). Western blot further validated that the levels of these UPR^mt^-related proteins were also elevated in Mut-H USCs (Fig. 4B–G). In addition, decreased expression of *WNT7B* (Fig. 4H), as well as decreased levels of p-GSK3β in Mut-H USCs were also confirmed (Fig. 4I, J).

### UPR^mt^ inhibition rescued defective mitochondrial functions in Mut-H USCs

**Fig. 5:**
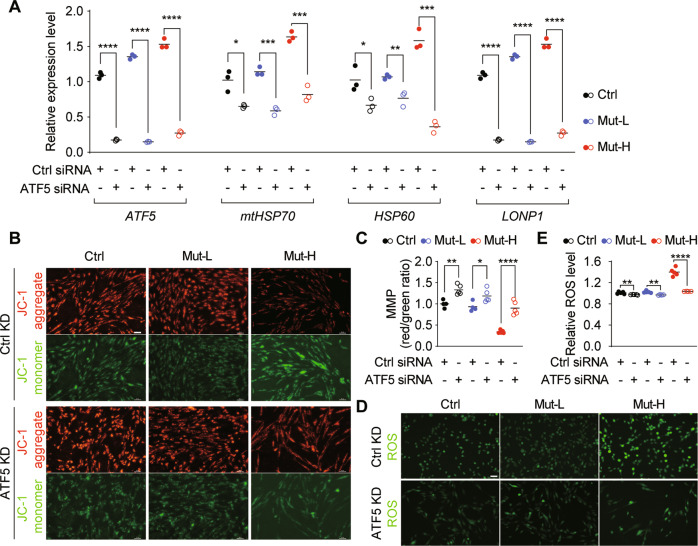
ATF5 knockdown reversed mitochondrial function in Mut-H USCs. **A** ATF5 knockdown decreased UPR^mt^ related gene expression. USCs were transfected with siRNA specific to *ATF5* for 48 h and the mRNA levels of UPR^mt^ related genes (*ATF5, HSP70, HSP60 and LONP1*) expression were quantified by RT-qPCR. **B** Mitochondrial membrane potential was increased by *ATF5* knockdown. USCs were transfected with siRNA specific to *ATF5* for 48 h and mitochondrial membrane potential was measured using fluorescence probe JC-1 assay system. Red color indicates cells with normal mitochondrial membrane potential while green indicates cells with loss of mitochondrial membrane potential. **C** Quantification plot for **B**. **D** Intracellular ROS was decreased by *ATF5* knockdown. USCs were transfected with siRNA specific to *ATF5* for 48 h and stained with DCFH-DA. Green color indicate ROS-positive cell population. **E** Quantification plot for **D**. *P < 0.05, **P < 0.01, ***P < 0.001, ****P < 0.0001. Scale bar = 50 um.

ATF5 is a critical transcription factor mediating UPR^mt^ in human cells [30]. In our study, upon *ATF5* knockdown by siRNA, the expression levels of *mtHSP70*, *HSP60* and *LONP1* were greatly reduced in all USCs (Fig. 5A), consistent with a critical role of ATF5 in regulating UPR^mt^. To further investigate the role of UPR^mt^ in m.3243A > G induced mitochondrial deficits, we examined *ΔΨ*_*m*_ and ROS after *ATF5* knockdown. *ATF5* deficiency increased *ΔΨ*_*m*_ (Fig. 5B, C), and reduced ROS levels (Fig. 5D, E) in all USCs. Importantly, the abnormally high ROS levels in Mut-H USCs were profoundly reduced (Fig. 5D, E), reaching wild-type levels. These results suggest that targeting ATF5-mediated UPR^mt^ could improve mitochondrial functions that are impaired by high level of the m.3243A > G mutation.

### UPR^mt^ inhibition alleviated deficits in osteogenesis from m.3243A > G USCs

**Fig. 6:**
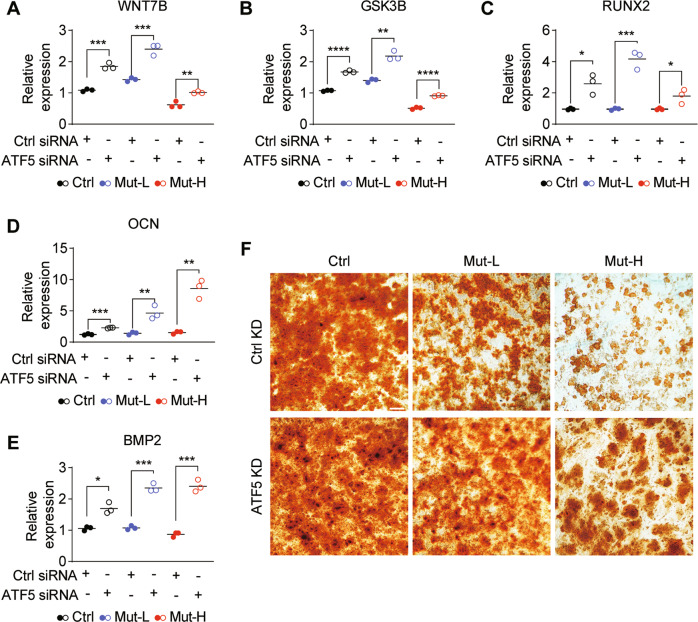
Osteogenesis was impaired in Mut-H USCs but could be alleviated by ATF5 knockdown. **A**, **B** *ATF5* knockdown increased *WNT7B* and *GSK3B* gene expression. *ATF5* was knockdown by siRNA for 48 h and the mRNA levels of *WNT7B* and *GSK3B* were quantified through RT-qPCR. **C**–**E** Expressions of osteogenesis-related markers were increased by *ATF5* knockdown in all USCs. USCs were transfected with siRNA specific to *ATF5* for 48 h and cultured in osteogenic induction medium for 48 h. mRNA levels of *RUNX2, OCN* and *BMP2* were quantified through RT-qPCR. **F** Alizarin Red S staining of calcium levels in Ctr, Mut-L, and Mut-H USCs. USCs were cultured in osteogenic induction medium with or without siRNA transfection specific to *ATF5* for 48 h and cultured in osteogenic induction medium for 21 days. Cells were stained with Alizarin Red S and imaged with dissecting microscope under bright field. *P < 0.05, **P < 0.01, ***P < 0.001, ****P < 0.0001. Scale bar = 50 µm.

Since inhibiting UPR^mt^ improved mitochondrial functions in Mut-H USCs (Fig. 5), given that ATF5 has been reported to play a regulatory role during osteogenesis [33], we examined whether inhibition of ATF5 could rescue the osteogenesis defect. Upon *ATF5* knockdown, the levels of *GSK3B* and *WNT7B* were increased in all USCs, and restored to wild-type levels in Mut-H USCs (Fig. 6A, B). *RUNX2*, *OCN* and *BMP2* are classic markers for osteogenesis. The osteogenesis defect was shown by immunocytochemical analyses as well as Alkaline Phosphatase and Alizarin Red S staining (Fig. S4). Knockdown of *ATF5* increased the expression of *RUNX2*, *OCN* and *BMP2* (Fig. 6C–E), suggesting enhanced osteogenic potentials. Further, *ATF5* knockdown corrected the osteogenesis defect in m.3243A > G Mut-H USCs (Fig. 6F), suggesting this gene may serve as a potential therapeutic target for treatment of osteoporosis related to m.3243A > G mutation.

## Discussion

In this study, we reported the first case of generating urine-derived mesenchymal stem cells (USCs) from m.3243A > G patients. We discovered that ATF5-mediated UPR^mt^ could be an essential mechanism underlying the impaired mitochondrial function and poor osteogenic potentials in Mut-H USCs. Our study represented one of many efforts to circumvent the hurdles in establishing appropriate in vitro models for mitochondrial mutation research. The lack of effective in vitro and in vivo models of mitochondrial DNA mutation has hampered mechanistic studies [35]. The traditional cytoplasmic hybrid (cybrid) cell lines [36] or human induced pluripotent stem (hiPS) cells were either artificial or laborious to generate [15, 37, 38]. HiPS cells often carry additional genetic and mitochrondral mutations created during the induction process [39, 40]. Recently, we and others have successfully derived USCs from human subjects through a simple, low-cost, and non-invasive method [41, 42]. One interesting feature of our USCs from patients with the m.3243A > G mutation was the bimodal distribution of heteroplasmy. Some clones were nearly homoplasmic wild-type while the majorities were nearly homoplasmic mutant-type. The mutant-high USCs could be an ideal in vitro model for studying the cellular and molecular alterations resulted from the m.3243A > G mutation, while mutant-low USCs could be potential cell sources for autologous cell-replacement therapy. Medium heteroplasmy is potentially linked to a growth disadvantage, which remains to be determined.

Our results provided supports to the emerging role of m.3243A > G in bone mineralization deficiency. We and another group recently reported that m.3243A > G mutation was associated with loss of bone density in affected patients [11, 12]. In the current study, we further confirmed this newly characterized pathological phenotype (Table 1), and started to unveil the underlying signaling pathways linking the m.3243A > G mutation to the loss of bone density. By taking advantages of the in vitro USCs model, we showed that activation of the mitochondrial stress response UPR^mt^ and the decline in the Wnt/β-catenin pathway could result in poor osteogenic potentials of Mut-H USCs. Inhibition of UPR^mt^ by knocking down of *ATF5* reversed the expression of *GSK3B* and *WNT7B*, resulting in increased mineralization of in vitro cultured Mut-H USCs. Our study has therefore suggested that ATF5 could be a novel and potential therapeutic target for treating osteoporosis due to m.3243A > G. The improved osteogenesis from *ATF5*-depleted USCs might be linked to improved mitochondrial functions. It remains to be determined whether other m.3243A > G-associated pathological phenotypes such as diabetes, deafness, and other symptoms in MELAS could also be ameliorated by *ATF5* targeted inhibition.

The upstream signaling turning on UPR^mt^ in mutant-high m.3243A > G remains unclear. UPR^mt^ can be activated by an imbalance between mitochondrial versus nuclear protein synthesis. Therefore, one possibility is that the m.3243A > G mutation to tRNA gene reduces protein synthesis in the mitochondria, triggering the mitonuclear imbalance, hence UPR^mt^ activation. Indeed, decreased mitochondrial translation in m.3243A > G cells has been reported in several studies [21, 22], and increased expression of mitochondrial stress-responsive genes including heat shock protein (HSPs) have also been reported [16]. Interestingly, mutant-low USCs did not induce UPR^mt^, nor did they have altered osteogenesis or mitochondrial morphology or functions. These results were somewhat expected, because the mutant-low USCs in this study have mutation rate <5%, which was not much different from wild-type cells.

The finding that UPR^mt^ played a negative role in mitochondrial function and osteogenesis was surprising. UPR^mt^ targeted genes involved in protein homeostasis and had been shown in many studies to serve as a positive modulator of mitochondrial function and promoted survival. Loss of critical regulator such as ATF5 and its homologs ATFS-1 compromised cell viability and reduced lifespan of *C. elegans*. However, two recent studies showed that UPR^mt^ could also function to promote the expansion of defective mtDNA, leading to accumulation of defective mitochondria, whereas UPR^mt^ inhibition preferentially depleted defective mtDNA [31, 32]. The UPR^mt^ could function similarly in our study, where UPR^mt^ activation in USCs kept high levels of m.3243A > G mutation, leading to compromised mitochondria and reduced osteogenesis. On the contrary, inhibition of UPR^mt^ by *ATF5* knockdown improved mitochondrial function and promoted osteogenesis, through mechanisms involving amplified Wnt signaling. It will be important to study at the molecular level, whether and how UPR^mt^ regulates m.3243A > G heteroplasmy in the disease contexts.

Taken together, by establishing human USCs as an in vitro model to study mitochondria DNA mutations, we revealed an important role of *ATF5*-dependent UPR^mt^ in maintaining mitochondrial functions and osteogenic potentials. Our study provided new insights for better understanding osteoporosis in patients with m.3243A > G mutation and identified ATF5 being a potential therapeutic target for this pathological condition.

## Materials and methods

### Human subjects

This study was approved by the Institutional Review Board of Shanghai Jiao Tong University Affiliated Sixth People’s Hospital and was conducted in accordance with the Declaration of Helsinki. Written informed consent was obtained from each subject. Participants were recruited to screen for the m.3243A > G mutation at the Shanghai Jiao Tong University Affiliated Sixth People’s Hospital. A total of 13 participants carrying the m.3243A > G mutation were found, including 12 carriers with diabetes and 1 carrier with normal glucose tolerance. In addition, thirteen sex- and age-matched healthy controls with no m.3243A > G mutation, no history of diabetes, no hearing difficulties and no osteoporosis were also enrolled. The general clinical characteristics of each participant (e.g., history of diabetes and complications, treatment, as well as mitochondria-associated symptoms) were obtained through standard questionnaires and comprehensive clinical examinations.

### m.3243A > G mutation analysis

Peripheral blood leukocytes, saliva, and urine samples were obtained from all subjects. DNA was extracted from samples using an automated nucleic acid extraction instrument (Lab-Aid 820; BioV, China). High-resolution melting analysis was used for rapid m.3243A > G mutation scanning. The accurate quantification of the heteroplasmy levels of the m.3243A > G mutation in different samples was determined by pyrosequencing as previously described [43].

### Cell culture

Urine samples from three m.3243A > G participants and three healthy individuals were collected and cultured. Isolation and amplification of USCs has been described before [34, 44]. Fresh urine samples (200 ml) from patients were processed immediately by adding penicillin (10 kU/ml) and streptomycin (10 mg/ml) to prevent contamination, then centrifuged and washed with phosphate-buffered saline (PBS). The sediment containing live cells was resuspended in Dulbecco’s modified Eagle medium (DMEM) supplemented with 2 % (vol/vol) fetal bovine serum (FBS; Gibco, USA), 10 ng/ml of human epidermal growth factor (hEGF), 2 ng/ml of platelet-derived growth factor (PDGF), 1 ng/ml of transforming growth factor (TGF)-β, 2 ng/ml of basic fibroblast growth factor (bFGF), 0.5 μM hydrocortisone, 25 μg/ml of insulin, 20 μg/ml of transferrin, 549 ng/ml of epinephrine, 50 ng/ml of triiodothyronine (T3), L-glu and antibiotics. The cell suspension was plated into gelatin-coated 96-well plates and incubated at 37 °C in a humidified atmosphere with 5 % CO_2_. After 7 days, nonadherent cells were removed by washing with PBS and colonies derived from single cells were obtained. The cells were passaged using 0.25% trypsin before confluence.

### Identification of USC surface markers by flow cytometry

The protocol to characterize cell surface markers by flow cytometry was modified [44]. Briefly, cells were blocked with cold PBS containing 1% bovine serum albumin (BSA) for 30 min, then incubated with the following fluorescence-conjugated antibodies (Becton Dickinson, USA) for 1 h: CD29-PE, CD73-PE, CD90-PE, CD44-FITC, CD13-FITC, SSEA4-PE, CD31-FITC, CD45-FITC, CD34-PE and HLA-DR-PE. Isotype-matched monoclonal antibodies were used as controls (BD Biosciences). Cells were washed to remove unbound antibodies and analyzed by using Guava easyCyte™ (Millipore, Billerica, MA, USA).

### Karyotype analysis

Karyotype analysis was used to test chromosomal stability of USCs at passage 9. Cells were incubated in 20 µg/ml colchicines for 4 h at room temperature and then treated with 0.075 mM potassium chloride for 15 min, finally fixed with methanol-to-acetic acid solution (3:1). Geimsa staining were performed to visualize G-banding. Images were captured by microscope (BX51; Olympus, Japan).

### RNA sequencing and library preparation

#### Total RNA extraction

Total RNA was extracted from the tissues using Trizol (Invitrogen, Carlsbad, CA, USA) according to manual instruction. About 60 mg of tissues were ground into powder by liquid nitrogen in a 2ml tube, followed by being homogenized for 2 min and rested horizontally for 5 min. The mix was centrifuged for 5 min at 12,000 × *g* at 4 °C, then the supernatant was transferred into anew EP tube with 0.3 ml chloroform/isoamyl alcohol (24:1). The mix was shacked vigorously for 15 s, and then centrifuged at 12,000 × *g* for 10 min at 4 °C. After centrifugation, the upperaqueous phase where RNA remained was transferred into a new tube with equal volume ofsupernatant of isopropyl alcohol, then centrifuged at 13,600 rpm for 20 min at 4 °C. Afterdeserting the supernatant, the RNA pellet was washed twice with 1 ml 75% ethanol, then the mix was centrifuged at 13,600 rpm for 3 min at 4 °C to collect residual ethanol, followed by thepellet air dry for 5-10 min in the biosafety cabinet. Finally, 25–100 µl of DEPC-treatedwater was added to dissolve the RNA. Subsequently, total RNA was qualified and quantified using a Nano Drop and Agilent 2100 bioanalyzer (Thermo Fisher Scientific, MA, USA).

#### mRNA library construction

Oligo(dT)-attached magnetic beads were used to purified mRNA. Purified mRNA was fragmentedinto small pieces with fragment buffer at appropriate temperature. Then First-strand cDNA wasgenerated using random hexamer-primed reverse transcription, followed by a second-strand cDNAsynthesis. afterwards, A-Tailing Mix and RNA Index Adapters were added by incubating to endrepair. The cDNA fragments obtained from previous step were amplified by PCR, and productswere purified by Ampure XP Beads, then dissolved in EB solution. The product was validated on the Agilent Technologies 2100 bioanalyzer for quality control. The double stranded PCR productsfrom previous step were heated denatured and circularized by the splint oligo sequence to get the final library. The single strand circle DNA (ssCir DNA) was formatted as the final library. The final library was amplified with phi29 to make DNA nanoball (DNB), which had >300 copies of one molecular, DNBs were loaded into the patterned nanoarray and single end 50 basesreads were generated on BGIseq500 platform (BGI-Shenzhen, China)

### Transmission electron microscopy (TEM)

Cells were fixed in 2.5% glutaraldehyde, and then loaded to copper grids coated with Formvar. After dehydration through a graded series of ethanol, the cell samples were embedded in Epon. Ultrathin sections were stained with 2% uranyl acetate and lead citrate, then examined by TEM (H-7650, HITACHI, Japan)

### ROS measurement

DCFH-DA probe was used for intracellular ROS measurement. Briefly, cells were washed with PBS and harvested, followed by incubation with 10 µM DCFH-DA at 37 °C for 30 min in the dark. Cells were then washed with PBS and resuspended in DMEM. Fluorescence intensity was detected by a Fluorescence Microscope (Nikon Ti-U, Tokyo, Japan) with excitation (Ex) and emission (Em) wavelengths of 488 and 525 nm and the images were obtained with a Nikon Digital Sight DS-Fi2 camera. All fluorescence intensities were measured by ImageJ. Cellular ROS contents were also measured using a Cytomix FC500 flow cytometer.

#### Mitochondrial membrane potential measurement

Tetra-ethyl-benz-imidazolyl-carbocyanine iodide (JC-1) was used to measure mitochondrial membrane potential as described before [45, 46]. USCs in DMEM were incubated with an equal volume of staining solution containing 5 μg/ml JC-1 at 37 °C for 20 min. Cells were washed three times with PBS and resuspended in DMEM. The samples were observed under a Fluorescence Microscope with Ex/Em wavelengths of 490/530 nm for JC-1 monomers showing green fluorescence and Ex/Em wavelengths of 525/590 nm for JC-1 aggregates showing red fluorescence. The ratio of red fluorescence and green fluorescence represented the *ΔΨ*_*m*_ of USCs. Fluorescence intensities were measured by ImageJ software [45]. The samples were also analyzed with the Cytomix FC500 flow cytometer [47]. Mitochondrial membrane potential abnormality was expressed as “increases in the percentage of abnormal-MMP cells”.

#### Real-time quantitative PCR (RT-qPCR)

Total RNA was isolated by using Trizol and reverse transcribed by using HiScript II Q RT SuperMix for qPCR (Vazyme, China). RT-qPCR was performed by using AceQ Universal SYBR qPCR Master Mix (Vazyme, China) with corresponding primer sets, which can be found in the supplemental Table S4. Gene expression levels were calculated using the 2^−ΔΔCt^ method (Livak and Schmittgen 2001). All assays were repeated at least three times.

### Western blotting

The total proteins were extracted from USCs using RIPA Lysis Buffer (Beyotime, Shanghai, China). Total cell lysates were separated on 10% sodium dodecyl sulfate polyacrylamide gel electrophoresis and transferred onto polyvinylidene fluoride membranes. After blocking with 5% nonfat dried milk in TBST, blots were probed with primary antibodies to ATF5, *mtHSP70*, *HSP60*, *LONP1*, β-actin (Abcam, USA. ATF5: ab184923, mtHSP70: ab171089, HSP60: ab190828, LONP1: ab103809, β-actin: ab8226) overnight at 4 °C and secondary antibody (Abcam, Cambridge, MA, USA) incubation at 37 °C for 1 h according to standard protocols. Immunoreactive proteins were visualized using an electrochemiluminescence system (Tinon, China).

### siRNA knockdown

The siRNAs used for downregulation of the capsid genes as well as the negative control siRNA were obtained from GenePharma, Shanghai, China (ATF5 siRNA: GCGAGUUUGAUUUCAAGCUTT, AGCUGUGAAAUCAACUCGCTT). The transient transfection of siRNA was performed with the siRNA-Mate transfection reagent (GenePharma) according to manufacturer’s instruction.

### Osteogenic differentiation and identification

When USCs reached 80% confluence, cells were induced to differentiated into osteogenic lineage cells by osteogenic induction media (Cyagen Biosciences, China). To investigate the effect of inductive osteogenesis by gene transfection only, ATF5 siRNA transduced USCs were cultured in osteogenic induction media at 37 °C and 5% CO_2_. The medium was replaced every two to three days. After 14 days induction, alkaline phosphatase staining was performed by an Alkaline Phosphatase Kit (Sigma-Aldrich, St. Louis, MO, USA) according to the manufacturer’s instructions. After 21 days induction, Alizarin Red S staining was utilized to detect calcified matrix deposition as described before [48]. Briefly, cells were washed with PBS and fixed with 4% formaldehyde solution for 10 min, then stained with 1% Alizarin Red S for 5 min. Cells were washed with PBS thoroughly to remove unbound dye and microscopic imaged.

### Statistical analysis

Data were expressed as the mean ± STD or as median (interquartile range 25–75%) as appropriate. Differences between m.3243A > G carriers and controls were determined using the Student’s *t*-test or the Mann–Whitney *U*-test. Differences among mutant-high, mutant-low and control were analyzed by one-way ANOVA with Tukey’s correction for multiple comparisons. All *P*-values were two-sided, and values of *P* < 0.05 were considered statistically significant (*P < 0.05, **P < 0.01, ***P < 0.001, ****P < 0.0001). All statistics were performed with GraphPad Prism 8.

## Supplementary information

SI information

SI figure1

SI figure 2

SI figure 3

SI figure 4
