# Supplementary material for: ATF5, a putative therapeutic target for the mitochondrial DNA 3243A > G mutation-related disease
Source: Cell Death Dis. 2021 Jul 14;12(7):701. doi: 10.1038/s41419-021-03993-1 (PMC8280182; doi:10.1038/s41419-021-03993-1)
Supplement: Supplementary file 1 — SI information [file 41419_2021_3993_MOESM1_ESM.pdf]

Supplemental tables and figures

Table S1 Clinical characteristics of the people with the m.3243A>G mutation and the controls

	Subjects with m.3243A>G mutation	Controls	P
Age	44.23±14.67	43.61±12.67	0.91
Men,n(%)	8(61.5)	8(61.5)	1
BMI	19.4 (17.95, 20.1)	22.5 (20.05, 24.93)	0.64
heteroplamsy in leukocytes,%	21.15±11.15	/	
heteroplamsy in saliver,%	28.06±12.93	/	
heteroplamsy in urine sediment,%	64.24±18.81	/	
Diabetes,n(%)	12(92.3)	/	

Age at diagnosis,years	40.75±14.50	/	
Diabetes duration, years	6.67±7.62	/	
Diabetes complications, n(%)	3 （23.08）	/	
HbA1c, %	7.8 (6.1,9.3)	5.7 (5.4, 5.8)	<0.001
Sensorineural hearing loss, n(%)	8(61.54)	/	
Better ear hearling level0.5-4khz,dB	42.12±24.70	/	
Osteopenia/osteoporosis,n(%)	7(53.85)	/	
Total hip(g/cm2)	0.87±0.11	0.99±0.12	0.017
Total spine(g/cm2)	1.05±0.19	1.1±0.17	0.11
Femoral neck(g/cm2)	0.82±0.1	0.92±0.16	0.09

Femoral stem(g/cm2)	1.04±0.13	1.18±0.16	0.02

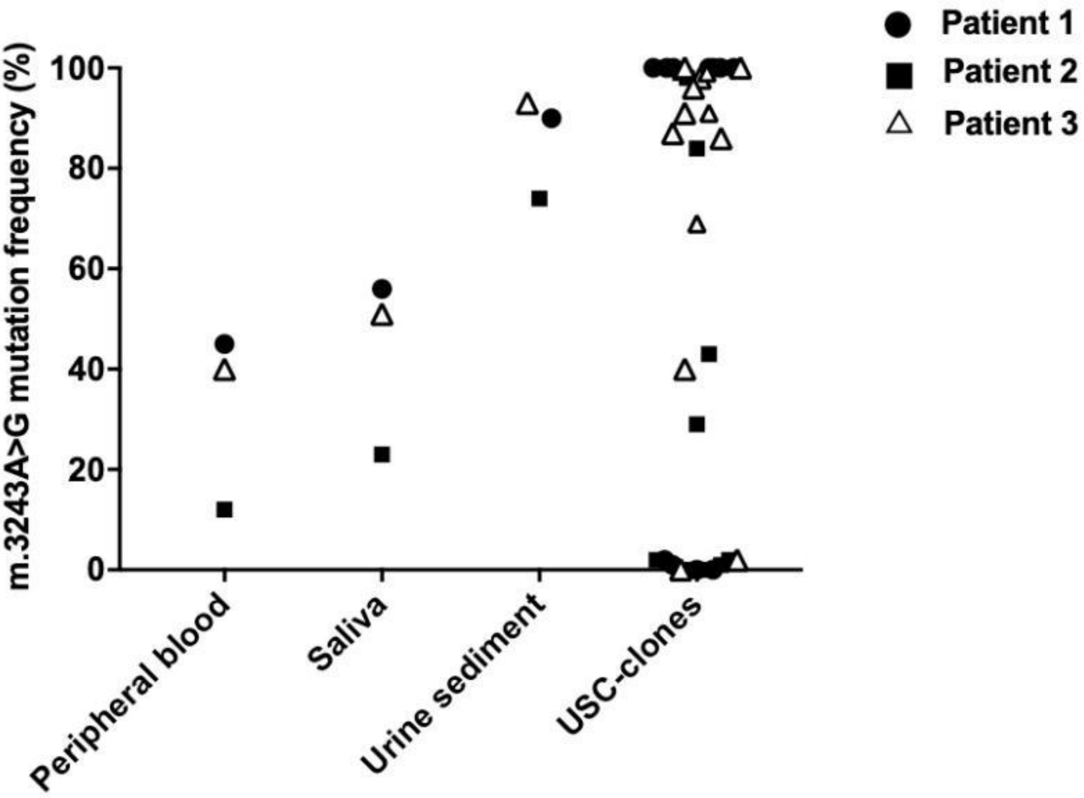

Figure S1. Distribution of m.3243A>G heteroplasmy levels in peripheral blood, saliva, urine sediment and USC clones derived from patients and relative proportion of Mut-H, Mut-Lclones in 3 different patients.

Table S2 Influence of cell culture and passaging on m.3243A>G mutation heteroplasmy of USCs clones

Hetero (%)	P1	P3	P5	P7
Mut-L 1	0	1	1	1
Mut-L 2	0	0	1	0
Mut-L 3	2	2	0	0
Mut-L 4	1	1	0	0
Mut-H 1	100	100	100	99
Mut-H 2	100	99	100	99
Mut-H 3	99	100	100	100
Mut-H 4	100	100	100	100

Hetero: heteroplasmy; P: passage

Table S3 m.3243A>G heteroplasmy levels of USCs sub-clones among the same passage

Hetero (%)	Mut-L 1	Mut-L 2	Mut-H 1	Mut-H 2
P2-1	0	0	100	100
P2-2	0	0	100	100
P2-3	0	0	98	100

Hetero: heteroplasmy; P2: passage 2 (all subclones were derived among passage 2)

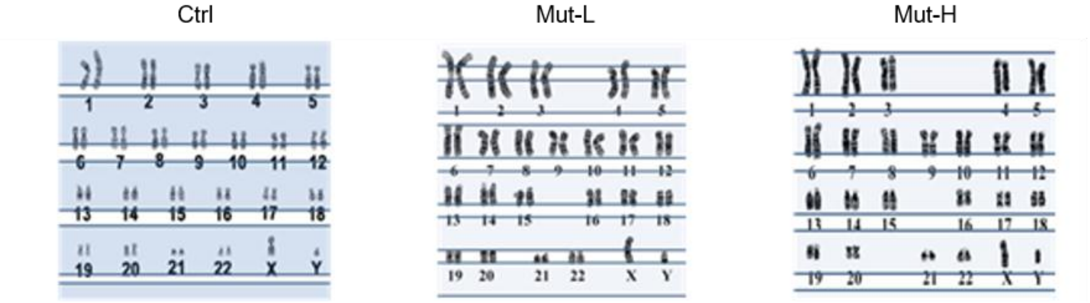

Figure S2 Karyotype analysis of m.3243A>G USCs.: Karyotype analysis of Ctrl, Mutant-low, and Mutant-high USCs at passage 9. USCs preserved its normal karyotypes and no obvious chromosomal rearrangements was found.

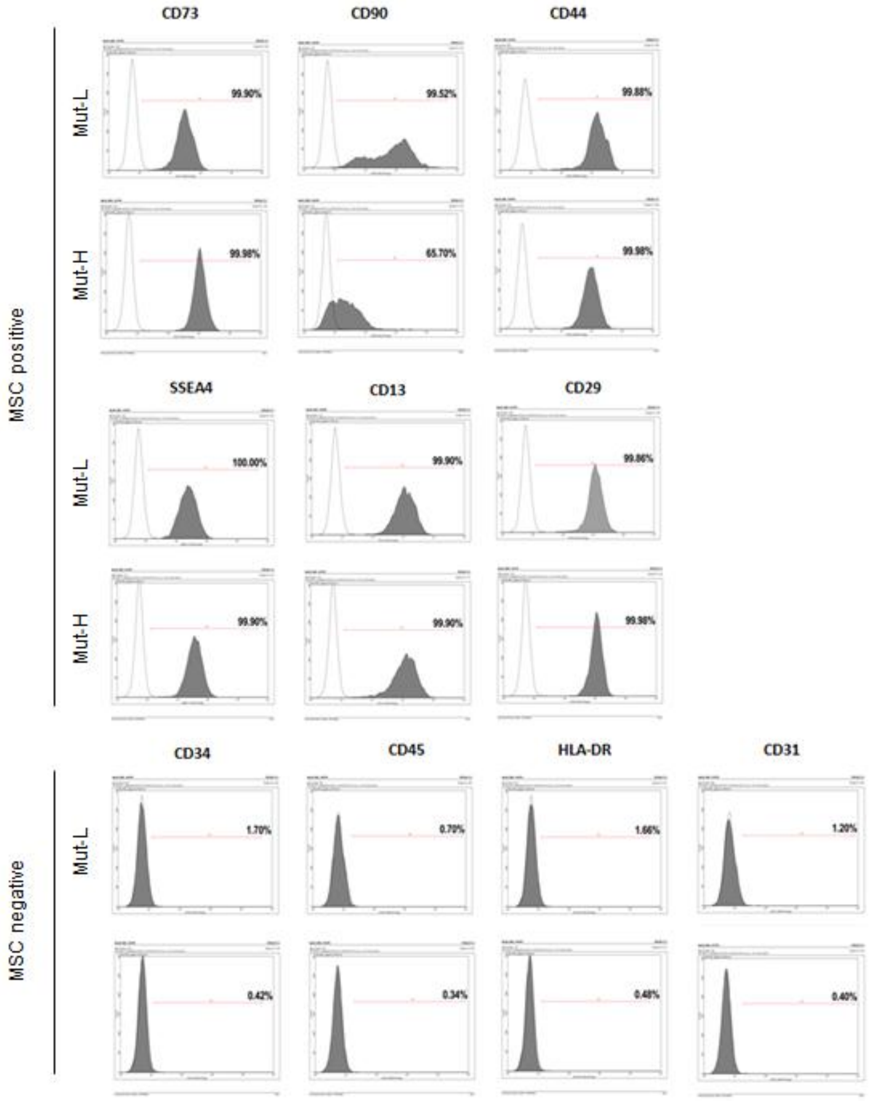

Figure S3 Characterization of cell surface markers confirmed mesenchymal stem cell origin. Indicated mesenchymal positive and negative markers were quantified by flow cytometry.

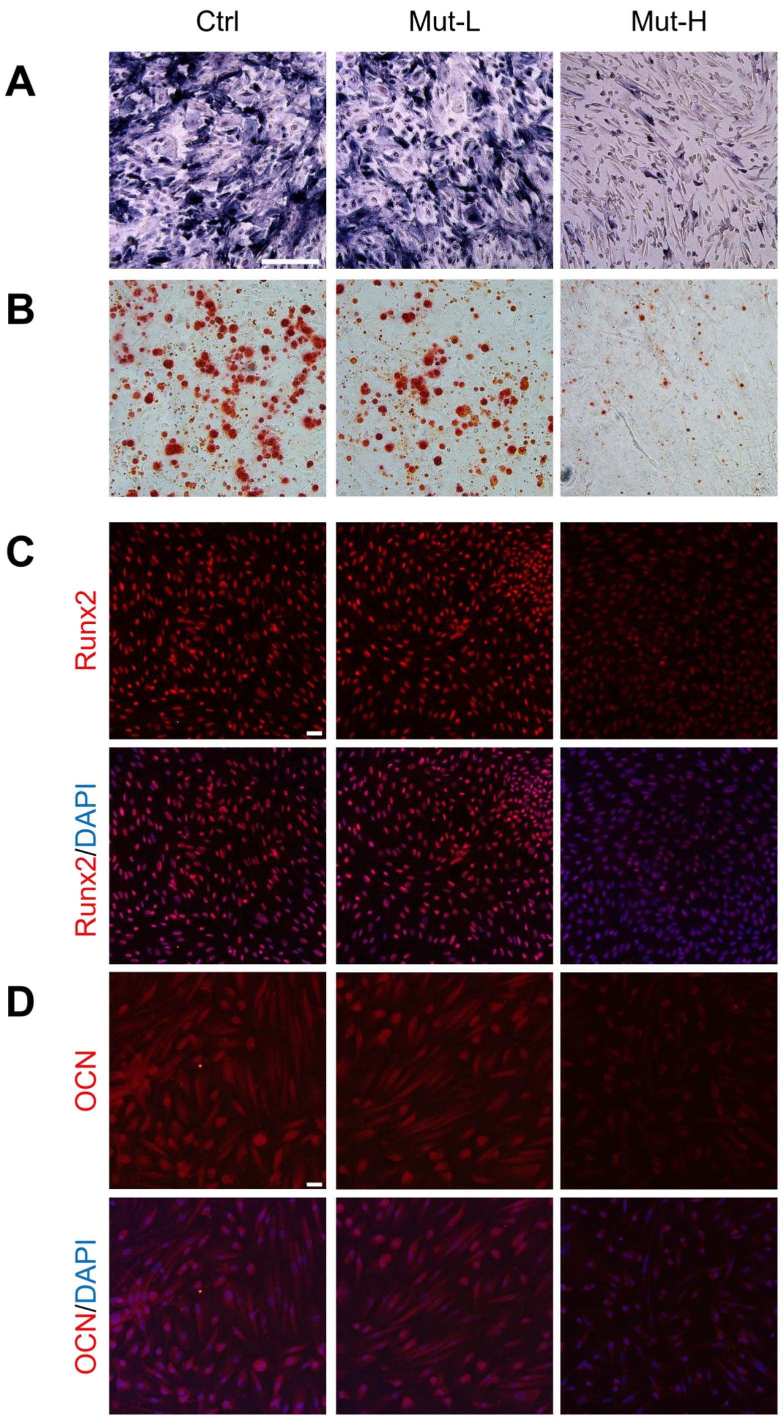

Figure S4 Osteogenic differentiation capability of m.3243A>G USCs. Mut-H USCs showed significantly decreased osteogenesis compared with Ctrl and Mut-L USCs. (A): ALP staining was performed at day 14; (B): Alizarin red staining for calcium deposition after 21 days induction. Scale bar = 250 um. (C-D): Immunofluorescence staining revealed expression of osteogenic-related proteins RUNX2 (C) and OCN (D) after 21 days of induction. Nuclear staining in blue (DAPI). Scale bar = 50 um.

Table S4 Primer sets for RT-qPCR

	Forward	Reverse
ATF5	CTGGCTCCCTATGAGGTCCTTG	GAGCTGTGAAATCAACTCGCTCAG
mtHSP70	CAAGCGACAGGCTGTCACCAAC	CAACCCAGGCATCACCATTGG
HSP60	GATGCTGTGGCCGTTACAATG	GTCAATTGACTTTGCAACAGTCACA C
Lonp1	CATTGCCTTGAACCCTCTC	ATGTCGCTCAGGTAGATGG
Runx2	CCAACCCACGAATGCACTATC	TAGTGAGTGGTGGCGGACATAC
BMP2	GAGAAGGAGGAGGCAAAGAAA	AGCAGCAACGCTAGAAGACAG
OCN	CCCCCTCTAGCCTAGGACC	ACCAGGTAATGCCAGTTTGC
GSK-3β	CCTTAACCTGGTGCTGGACT	AGCTCTGGTGCCAGTA
Wnt-7b	CAACGAGTGCCAGTACCAGTTCC	ATCTCCCGAGCGTCCACGAAG
β-actin	CATGTACGTTGCTATCCAGGC	CTCCTTAATGTCACGCACGAT
