# Supplementary figures and images for: ATF5, a putative therapeutic target for the mitochondrial DNA 3243A > G mutation-related disease
Source: Cell Death Dis. 2021 Jul 14;12(7):701. doi: 10.1038/s41419-021-03993-1 (PMC8280182; doi:10.1038/s41419-021-03993-1)

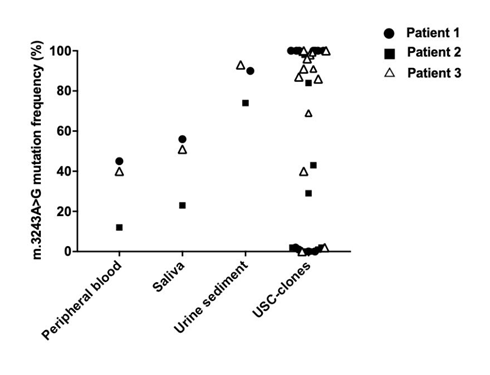

Supplement: Supplementary file 2 — SI figure1 [file 41419_2021_3993_MOESM2_ESM.tif]

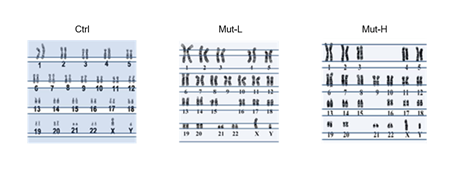

Supplement: Supplementary file 3 — SI figure 2 [file 41419_2021_3993_MOESM3_ESM.tif]

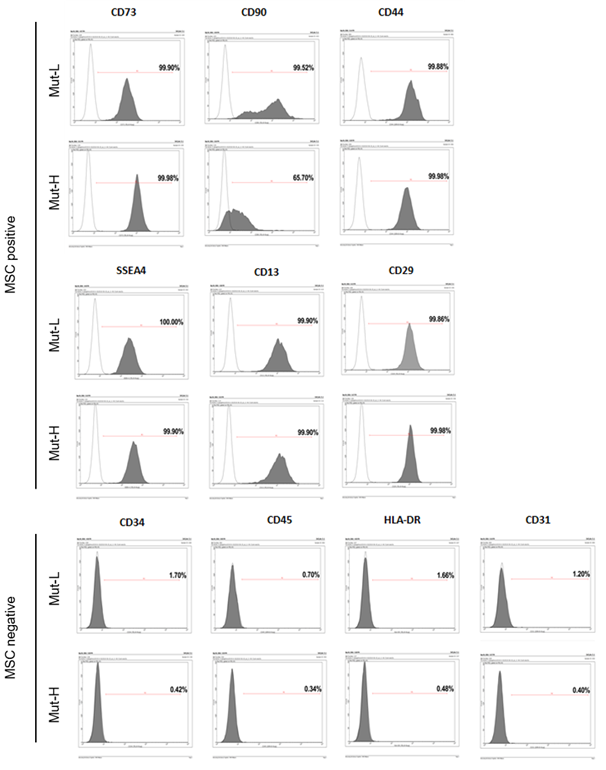

Supplement: Supplementary file 4 — SI figure 3 [file 41419_2021_3993_MOESM4_ESM.tif]

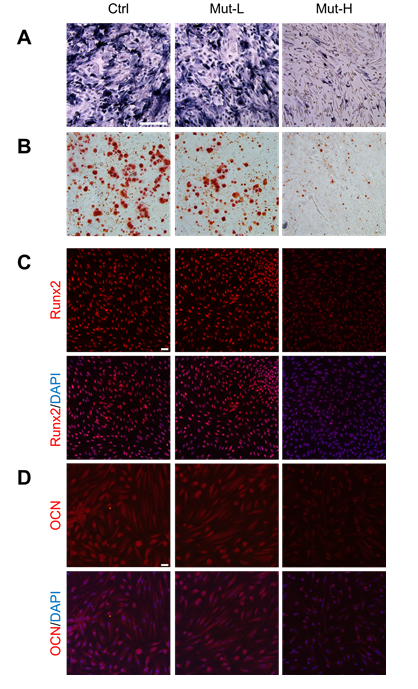

Supplement: Supplementary file 5 — SI figure 4 [file 41419_2021_3993_MOESM5_ESM.tif]
